# APPLYING NEW METHODS TO EXISTING DATASETS TO TEST STRESS EFFECTS ON BIOLOGICAL AGING

**DOI:** 10.1093/geroni/igac059.1466

**Published:** 2022-12-20

**Authors:** Daisy Zavala, Natalie Dzikowski, Shyamie Gopalan, Kerry Reid, Jennifer Graham-Engeland, Christopher Engeland, Krishna Veeramah, Stacey Scott

**Affiliations:** Stony Brook University, Queens, New York, United States; Stony Brook University, Stony Brook, New York, United States; Duke University, Durham, North Carolina, United States; Stony brook University, Stony Brook, New York, United States; Pennsylvania State University, Pennsylvania State University, Pennsylvania, United States; Pennsylvania State University, University Park, Pennsylvania, United States; Stony brook University, Stony Brook, New York, United States; Stony Brook University, Stony Brook, New York, United States

## Abstract

Novel opportunities to examine how life experiences connect to health and mortality through biological aging have increased due to the affordability of genomic analysis. Although epidemiological studies suggest associations between stress and accelerated biological aging, most if not all measures in the past have been global, data cross-sectional, and from primarily white samples. As part of a completed measurement study, a diverse sample (n=140, 25-65 years, Mage=47, 65% Female) provided detailed stress measures, including EMA and lifetime stress assessments, and consented to future analysis of their blood samples. We successfully used decade-old blood samples to estimate methylation-based epigenetic clocks and examined them within the context of various stress measures as a preliminary phase of a future longitudinal study. Additional whole genome sequencing will also allow for future work that incorporate continuous measures of genomic ancestry when examining lifetime discrimination-related stress, rather than simply testing for demographic group differences.

